# Re-emergence of hereditary polyneuropathy in Scandinavian Alaskan malamute dogs—old enemy or new entity? A case series

**DOI:** 10.1186/s13028-017-0295-y

**Published:** 2017-05-02

**Authors:** Karin Hultin Jäderlund, Cecilia Rohdin, Mette Berendt, Øyvind Stigen, Merete Fredholm, Arild Espenes, Inge Bjerkås, Lars Moe

**Affiliations:** 1 Department of Companion Animal Clinical Sciences, Faculty of Veterinary Medicine, Norwegian University of Life Sciences, P.O. Box 8146, 0033 Oslo, Norway; 2 Department of Clinical Sciences, Swedish University of Agricultural Sciences, Uppsala, Sweden; 3 Anicura, Albano Small Animal Hospital, Danderyd, Sweden; 4 Department of Veterinary Clinical Sciences, Faculty of Health and Medical Sciences, University of Copenhagen, Copenhagen, Denmark; 5 Department of Basic Sciences and Aquatic Medicine, Faculty of Veterinary Medicine, Norwegian University of Life Sciences, Oslo, Norway

**Keywords:** Polyneuropathy, Inherited, Dog, *NDRG1*-gene, Recessive, Mutation

## Abstract

A homozygous mutation has been identified in the N-myc downstream-regulated gene 1 (*NDRG1*) in recent cases of polyneuropathy in Alaskan malamute dogs from the Nordic countries and USA. The objective of the present study was to determine if cases diagnosed 30–40 years ago with polyneuropathy in the Alaskan malamute breed in Norway had the same hereditary disease as the recent cases. Fourteen historical cases and 12 recently diagnosed Alaskan malamute dogs with hereditary polyneuropathy, and their parents and littermates (n = 88) were included in this study (total n = 114). After phenotyping of historical and recent cases, *NDRG1* genotyping was performed using DNA extracted from archived material from five Norwegian dogs affected by the disease in the late 1970s and 1980s. In addition, pedigrees were analysed. Our study concluded that historical and recent phenotypic polyneuropathy cases were carrying the same *NDRG1*-mutation. The pedigree analysis showed that all affected Alaskan malamute cases with polyneuropathy could be traced back to one common ancestor of North American origin. By this study, a well-documented example of the silent transmission of recessive disease-causing alleles through many generations is provided, demonstrated by the re-emergence of a phenotypically and genetically uniform entity in the Scandinavian Alaskan malamute population.

## Findings

Degenerative polyneuropathies in dog breeds were reviewed in 2011 [1]. For most breeds, the disorder was considered inherited, with a breed-specific phenotype. Charcot-Marie-Tooth disease (CMT) constitutes the corresponding group of inherited polyneuropathies in humans. Common for canine polyneuropathies and the CMTs are dysfunctional peripheral nerves. The subtypes of inherited polyneuropathies may differ in characteristic details, e.g. clinical course, nerve fibre types involved, mode of inheritance and underlying gene mutation.

Some genetic aetiologies for canine polyneuropathies have recently been identified [2–6]. In humans, 80 different genes have been associated with CMT [7]. Some CMT-causing mutations are associated with considerable phenotypic variability and, conversely, a specific neurologic phenotype can apparently be caused by mutations in different genes, as also described in a canine breed [5, 8–10]. These confusing genotype-phenotype relationships are poorly understood, but might result from modifying genes, epigenetics or environments.

A hereditary polyneuropathy in the Alaskan malamute (HPAM) population in Norway, with an autosomal recessive mode of inheritance, was reported in the 1980s [11–13]. After advice from the researchers, the Norwegian Alaskan malamute (AM) club initiated a breeding strategy to exclude obligate or probable carrier dogs from breeding, and thereafter no cases were diagnosed for more than 20 years. However, recently cases of HPAM recurred in Scandinavia. In 2013, recent HPAM cases from the Nordic countries and USA were reported to be homozygous for a mutation in the N-myc downstream-regulated gene 1 (*NDRG1*) [4].

Breeding strategies against recessively inherited canine diseases purely based on phenotypic characteristics have also failed previously, e.g. as with the fox terrier hereditary ataxia [14] since, inadvertently, carrier animals can go undetected in the population. Nonetheless, based on phenotypic similarities one cannot decide whether the recurring entity is the same disease genetically. Furthermore, even if the same genotype was confirmed in affected animals from different periods, it is best to prove that the mutated allele has been transmitted silently through the generations by identifying a common ancestor for all affected cases, if possible. The alternative would be that a separate mutation had occurred in the same locus in parallel in another founder—while feasible, this is extremely unlikely.

Thus, one aim of the present study was to investigate if the genotype in archived tissue from historical Norwegian HPAM cases was the same as the genotype found in recent Scandinavian cases. We also aimed at linking the families of historical and recent Scandinavian cases together by pedigree analysis.

Altogether, 114 privately owned AM dogs were included in this case series. A total of eight historical DNA samples were available (only seven ultimately provided useable DNA for *NDRG1* testing); DNA samples from 21 recent dogs were previously *NDRG1* tested. Forty-one historical dogs were included in the pedigree only (they were not *NDRG1* tested) and of those, clinical information allowing a diagnosis of polyneuropathy was available for nine dogs. No ethics approval was required as samples were collected in accordance with the institutional guidelines for animal welfare and ethics and all diagnostic procedures and samplings would anyway have been carried out as part of the normal clinical diagnostics in affected animals.

**Fig. 1:**
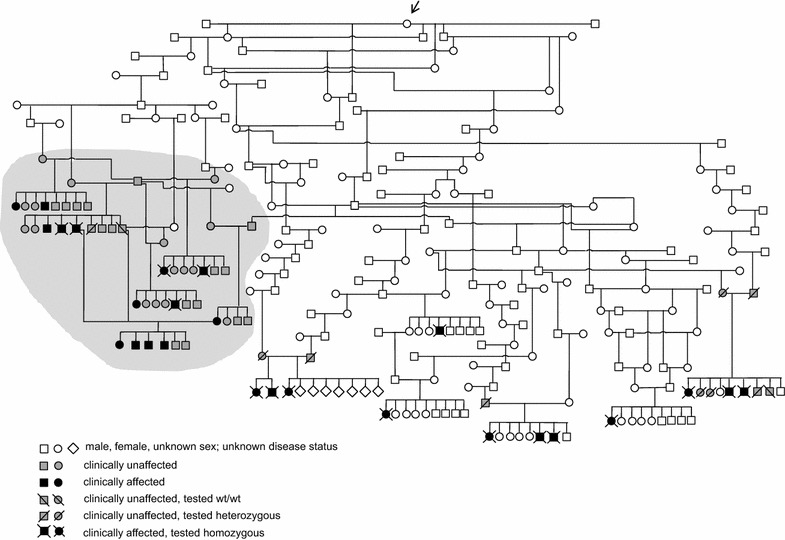
A schematic pedigree of Alaskan malamute dogs affected by hereditary polyneuropathy. A schematic pedigree of historical Norwegian and recent Scandinavian Alaskan malamute dogs affected by hereditary polyneuropathy, showing that both parents of all affected dogs are descending from one common ancestor. The families of historical dogs have a* shaded* background in the figure. Genotyped dogs are indicated as homozygous or heterozygous for the *NDRG1*-mutation, or homozygous wild type (wt/wt). Dogs that are neither affected nor genotyped are classified as either clinically unaffected or with unknown disease status. The mother of a test-mating litter of the historical dogs was inseminated. By accident, one straw of semen from another dog than the one meant to be father was used together with straws from the intended male, which is shown as two fathers for that specific litter in the figure. *Black arrow* points at common ancestor, born approximately 1955

Fourteen historical AM dogs with a clinical diagnosis of polyneuropathy, born in Norway from 1975 to 1983, were investigated (Fig. 1). Information characterizing the historical dogs was retrieved from archived clinical records, pedigrees, photos, histopathological specimens, electron microscopy images and images from muscle histochemistry. One of the authors (LM) had examined all historical cases clinically and neurologically at the Norwegian School of Veterinary Science (NVH). Predominant clinical signs were exercise intolerance, inspiratory stridor and pelvic limb ataxia. Nine of the cases were necropsied at NVH by one of the authors (IB). For the genotyping, archived formalin-fixed paraffin-embedded (FFPE) tissue blocks from six affected dogs were available.

In addition, archived information about all littermates (n = 27) and parents (n = 7) of the affected historical dogs were scrutinized. Four parents and four littermates had been examined at NVH, none of these showed signs of polyneuropathy. According to breeders and owners at that time, the other littermates (n = 23) and parents (n = 3) also appeared unaffected. For additional genotyping of historical material, archived frozen semen from one unaffected littermate and FFPE tissue blocks from another unaffected littermate were available. Both these dogs belonged to the group of littermates that were clinically examined at NVH.

Twelve affected recent dogs, born from 2001 to 2013, were from Norway (n = 9), Sweden (n = 2) and Denmark (n = 1). All had been tested homozygous for the *NRDG1*-mutation. Predominant clinical signs were exercise intolerance, inspiratory stridor and pelvic limb ataxia. They were clinically diagnosed with AM polyneuropathy, as described by Bruun et al. [4]. Five of them were also included in that study [4].

Furthermore, we included available information on parents and littermates (n = 54) of recent cases. Finally, we classified the littermates of all affected dogs as either “examined and unaffected” or “unknown clinical status”, as well as “heterozygous” or “wild type” for unaffected dogs genetically tested (Fig. 1).

Accordingly, tissues from altogether eight historical dogs were available for genotyping. DNA was extracted from FFPE tissue (n = 7) and frozen semen (n = 1) using a commercial purification kit (MasterPure™ DNA Purification Kit; Epicentre, Madison, USA) and phenol–chloroform extraction respectively. Genotyping was performed using a TaqMan assay as described previously [4].

Pedigrees of the recent (n = 12) and historically (n = 14) affected Scandinavian cases were retrieved from the Kennel Clubs of Norway, Denmark and Sweden and supplemented with ancestral information of relatives from the Kennel Clubs of America, Finland, Australia and the AM database. Pedigrees were compared, and lineages of both parents of affected dogs were traced back in time to the most recent common ancestor.

Extraction of DNA from tissue was successful in five historical cases and in the two unaffected littermates. The five cases were all homozygous for the *NDRG1*-mutation. One genotyped littermate was heterozygous and the other genotyped littermate was homozygous for the wild type allele (Fig. 1). Thereby, we could confirm that those historical cases had the same genetic background for their disorder as previously reported recent cases [4].

From the pedigree analyses it appeared that all affected dogs, recent and historical, had a North American female dog born approximately 1955 as their most recent common ancestor on both their father’s and their mother’s sides (Fig. 1). This points to her (or potentially one of her ancestors) as a possible and probable founder of the mutation. However, no DNA was available to prove this assumption.

The historical Norwegian and the recent Scandinavian HPAM-cases are linked together by having the same genotype and a common ancestry. HPAM, emerging in the Scandinavian population of AMs decades apart, should be considered one uniform entity. On a more general level, this re-emergence of a phenotypically and genetically uniform entity in the Scandinavian Alaskan malamute population provides a well-documented example of the silent transmission of recessive disease-causing alleles through many generations.

The breeding strategy by the Norwegian breed club aimed to exempt dogs from breeding that: (1) with certainty were carriers of the abnormal gene (parents: heterozygotes; and affected animals: homozygotes) and (2) to avoid using animals with a high probability of being carriers; (i.e. siblings of affected animals and offspring of parents that earlier had produced affected dogs). It was recommended (3) to record all information about HPAM status in future offspring and (4) to demonstrate a supportive attitude to breeders who unintentionally had produced HPAM cases.

The breeding strategy of the Norwegian AM club was indeed successful in preventing new cases of HPAM among descendants of the families directly involved in the historical cases (Fig. 1). The return of HPAM in Scandinavia was still not surprising, given the mode of inheritance and the mobility of dogs between different countries. As recessive alleles can be transmitted silently through generations, unintentional mating of heterozygotes to each other, resulting in affected homozygotes, was to be anticipated. The disease-causing mutation seems to have segregated in the breed for at least 60 years and, on a world-wide basis, obviously there were other lineages in parallel in this breed, transmitting the mutation in the 1970s and 1980s.

A breeding strategy like the one applied in Norway for HPAM puts a negative pressure on the total gene pool of the involved population. By narrowing the gene pool in a breed, there is a risk of more breed-related health problems as a consequence to increasing frequency of other defective genes. For HPAM, a genetic test is now available [4] and is useful for identification of carriers of the disease. By this reliable new tool, strategic use of heterozygous individuals for breeding, and eventually eradication of the mutation from the breed is possible.

In conclusion, HPAM is an inherited autosomal recessive disease, caused by the same mutation in the *NDRG1* in historical and recent Scandinavian cases.

## Abbreviations

AM: Alaskan malamute
FFPE: Formalin-fixed paraffin-embedded
HPAM: Hereditary polyneuropathy in the Alaskan malamute
NDRG1: N-myc downstream-regulated gene 1
NVH: The Norwegian School Of Veterinary Science
